# Postoperative outcomes following preoperative inspiratory muscle training in patients undergoing open cardiothoracic or upper abdominal surgery: protocol for a systematic review

**DOI:** 10.1186/2046-4053-1-63

**Published:** 2012-12-18

**Authors:** Christina M Mans, Julie C Reeve, Catherine A Gasparini, Mark R Elkins

**Affiliations:** 1 Physiotherapy Department, Waikato Hospital, Waikato District Health Board, Hamilton, New Zealand; 2 Division of Rehabilitation and Occupation Studies, Faculty of Health and Environmental Studies, AUT University, Auckland, New Zealand; 3 Disability Service, Student Support Services, University of Western Sydney, Sydney, Australia; 4 Centre for Evidence-Based Physiotherapy, The George Institute for Global Health, Sydney, Australia

**Keywords:** Inspiratory muscle training, preoperative, postoperative complications

## Abstract

**Background:**

In patients undergoing open cardiothoracic and upper abdominal surgery, postoperative pulmonary complications remain an important cause of postoperative morbidity and mortality, impacting upon hospital length of stay and health care resources. Adequate preoperative respiratory muscle strength may help protect against the development of postoperative pulmonary complications and therefore preoperative inspiratory muscle training has been suggested to be of potential value in improving postoperative outcomes.

**Methods/Design:**

A systematic search of electronic databases will be undertaken to identify randomized trials of preoperative inspiratory muscle training in patients undergoing elective open cardiothoracic and upper abdominal surgery. From these trials, we will extract available data for a list of predefined outcomes, including postoperative pulmonary complications, hospital length of stay and respiratory muscle strength. We will meta-analyze comparable results where possible, and report a summary of the available pool of evidence.

**Discussion:**

This review will provide the most comprehensive answer available to the question of whether preoperative inspiratory muscle training is clinically useful in improving postoperative outcomes in patients undergoing cardiothoracic and upper abdominal surgery. It will help inform clinicians working in the surgical arena of the likely effectiveness of instituting preoperative inspiratory muscle training programs to improve postoperative outcomes.

## Background

In patients undergoing open thoracic and upper abdominal surgery, changes in lung function are seen as being inevitable [1,2]. Patients develop predictable pulmonary changes that include altered respiratory mechanics, reduced lung volumes, respiratory muscle dysfunction and alterations in oxygenation status. These changes may be transient and self-resolving or may predispose patients to the development of more substantial complications. Postoperative pulmonary complications (PPCs) have been defined as ‘…a pulmonary abnormality that produces identifiable disease or dysfunction that is clinically significant and adversely affects the clinical course’ [2]. Consequences of PPCs include significant increases in length of hospital stay, patient discomfort, use of resources and overall hospital costs [3]. More importantly, PPCs are an important cause of postoperative morbidity and mortality following all types of major surgery [4,5].

Over the past two decades, widespread developments in the identification and modification of risk factors, patient education, surgical and anesthetic techniques, pain management and postoperative rehabilitation have led to reductions in postoperative complications following major surgery and faster discharge from hospital [6-9]. Despite this, PPCs continue to place a burden on health resources, particularly as surgery is now being offered to patient groups perceived to be at higher risk, such the elderly and those with comorbidities or more severe disease progression [10].

More recently, the focus on strategies to reduce postoperative complications and improve postoperative health-related quality of life has shifted to include prehabilitation. Prehabilitation may be described as the process of improving the functional capacity of the individual prior to a planned intervention, commonly surgery, to enable the individual to withstand the anticipated cardiovascular, respiratory, neuromuscular or musculoskeletal stressors [11]. Whilst currently the concept of prehabilitation and the evidence for its effectiveness are in their infancy, the main aims of prehabilitation are to improve postoperative outcomes and reduce postoperative risk. Those studies that have investigated prehabilitation have used a variety of preoperative exercise interventions across a spectrum of clinical settings, including joint replacement surgery and cardiac, thoracic and abdominal surgery [12]. In addition, the concept of prehabilitation to improve functional capacity before and after anticipated admission to intensive care has also been considered [11].

Some evidence suggests that adequate preoperative respiratory muscle strength and the ability to generate sufficient lung volumes may be protective against the development of PPCs [10,13]. Hence, preoperative training of the inspiratory muscles is one of several prehabilitation interventions currently coming under increasing investigation. Inspiratory muscle training (IMT) is a technique that targets the muscles of inspiration and aims to increase inspiratory muscle strength and endurance by applying an increased load to inspiration [14].

IMT can be undertaken in several ways including isocapnic/normocapnic hyperpnoea training, inspiratory resistive flow training and inspiratory threshold pressure training [15]. With isocapnic/normocapnic hyperpnoea, the patient breathes at a high percentage of maximum voluntary ventilation while maintaining isocapnia via entrainment of CO_2_ into the circuit. Isocapnic/normocapnic hyperpnoea allows the generation of high flow load with minimal pressure load and is used as respiratory muscle endurance training. Because isocapnic/normocapnic hyperpnoea involves a complex breathing circuit, is relatively time-consuming and is physically demanding, it is seldom used clinically, however, the development of a portable device for normocapnic hyperpnoea may increase its use in clinical trials [16]. Inspiratory resistive flow training refers to the technique of providing load by inspiring through a small aperture. One limitation of inspiratory resistive flow training is that the resistance provided may alter if the patient varies the inspiratory flow rate. Thus careful monitoring and feedback is required to ensure consistency of training loads. This problem of variable flow rates is solved by inspiratory threshold pressure devices where a spring- or plunger-loaded valve within the device opens to allow airflow when a predetermined threshold negative pressure is achieved during inspiration. Thus reproducible pressures can be assured [15]. Consensus recommendations regarding load, repetition and duration of training programs have not been established and may differ depending on the population of interest but, nonetheless, inspiratory muscles are known to respond to training in the same way as other skeletal muscles [17].

IMT has been shown to increase inspiratory muscle strength in healthy volunteers [18,19] and several patient populations, including patients weaning from mechanical ventilation [20], patients presenting with chronic lung disease [21] and patients with chronic heart failure [22]. As respiratory muscle dysfunction, particularly of the diaphragm, has been widely linked with the development of PPCs following major surgery [23,24], it is conceivable that strengthening the inspiratory muscles prior to surgery may impact upon postoperative recovery and reduce incidence of postoperative complications. This may shorten length of stay, reduce associated costs and improve patient outcomes.

Two recent systematic reviews are available that have estimated the effects of preoperative IMT (amongst a range of preoperative interventions) in patients undergoing major surgery [12,14]. However, the estimates of the effects of IMT may be affected by the methods used in these reviews. Olsén and Anzén [14] considered a range of outcomes but did not attempt a meta-analysis. Conversely, Valkenet and colleagues [12] performed a meta-analysis for the outcome PPCs, but did not consider important outcomes like oxygenation, mortality, quality of life, adverse events and costs. Also, both reviews excluded studies because of the language of publication and did not attempt to contact authors for unpublished data. Furthermore, since the searches were conducted for these reviews, additional data has been published (for example [25,26]), therefore a comprehensive review of this topic is appropriate.

The aims of this systematic review are to answer these questions: 

• Does preoperative IMT increase inspiratory muscle strength and endurance in patients undergoing open cardiothoracic or upper abdominal surgery?

• Does preoperative IMT reduce the incidence of PPCs and length of hospital stay in these patients?

• Does preoperative IMT affect the duration of mechanical ventilation, lung function, oxygenation, time to first sit out of bed and to first ambulation, exercise tolerance, adverse events, quality of life, mood, satisfaction, mortality and costs?

## Methods/Design

### Inclusion criteria for studies in the review

Types of studies: Eligible studies will be randomized controlled trials and quasi-randomized controlled trials.

Types of participants: Eligible participants will be adults (16 years and over) who are undergoing any elective open cardiac, thoracic or upper abdominal surgery, defined as surgery with an incision above or extending above the umbilicus [22,27].

Types of interventions: Preoperative IMT including isocapnic/normocapnic hyperpnoea, inspiratory resistive flow training (for example, with Pflex brand) or threshold pressure loading (for example, with Threshold brand) compared to sham or no IMT.

Primary outcomes:

1. Rates of PPCs (as defined by the individual studies)

2. Length of postoperative hospital stay (or total inpatient stay if this is what is reported)

Secondary outcomes

1. Measures of respiratory muscle strength, such as maximal inspiratory pressure (MIP) measured from residual volume and maximal expiratory pressure measured from total lung capacity

2. Measures of inspiratory muscle endurance, such as sustained MIP and maximal threshold load

3. Measures of exercise tolerance as measured by a standardized test (such as six minute walk test)

4. Lung volumes as measured by spirometry, such as forced vital capacity, forced expiratory volume in one second and vital capacity

5. Duration of postoperative mechanical ventilation

6. Measures of oxygenation

7. Postoperative mortality

8. Time to first sit out of bed

9. Time to first ambulation

10. Health-related quality of life

11. Measures of anxiety and depression

12. Measures of patient satisfaction

13. Adverse events

14. Costs

### Search strategy

The following electronic databases will be searched for all available years: Medline, CINAHL, AMED, PsycINFO, Scopus, PEDro and the Cochrane Library (specifically the Cochrane Database of Systematic Reviews, and the Cochrane Central Register of Controlled Trials). The search will not be limited by date, language or publication status. We will check the reference lists of any eligible studies identified for further relevant studies. We will also ask authors of eligible trials and manufacturers of IMT devices if they know of other eligible studies.

EBSCOhost Health Databases Search Strategy (Medline, CINAHL, AMED and PsychINFO):

1. (inspirat* N3 train*) OR (inspirat* N3 condition*) OR (respirat* N3 train*) OR (respirat* N3 condition*) OR (ventil* N3 train*) OR (ventil* N3 condition*) OR (pulmonary N3 train*) OR (pulmonary N3 condition*) OR (breath* N3 train*) OR (breath* N3 condition*)

2. preoperative OR pre-operative OR presurg* OR pre-surg*

3. #1 AND #2

Scopus Search Strategy:

1. (inspirat* W/3 train*) OR (inspirat* W/3 condition*) OR (respirat* W/3 train*) OR (respirat* W/3 condition*) OR (ventil* W/3 train*) OR (ventil* W/3 condition*) OR (pulmonary W/3 train*) OR (pulmonary W/3 condition*) OR (breath* W/3 train*) OR (breath* W/3 condition*)

2. preoperative OR pre-operative OR presurg* OR pre-surg*

3. #1 AND #2

PEDro Search Strategy:

1. inspirat musc train in Abstract & Title field

2. respirat musc train in Abstract & Title field

3. inspirat musc condition in Abstract & Title field

4. respirat musc condition in Abstract & Title field

*Cochrane Search Strategy (*via *Wiley):*

1. (inspirat* NEAR/3 train*) OR (inspirat* NEAR/3 condition*) OR (respirat* NEAR/3 train*) OR (respirat* NEAR/3 condition*) OR (ventil* NEAR/3 train*) OR (ventil* NEAR/3 condition*) OR (pulmonary NEAR/3 train*) OR (pulmonary NEAR/3 condition*) OR (breath* NEAR/3 train*) OR (breath* NEAR/3 condition*)

2. preoperative OR pre-operative OR presurg* OR pre-surg*

3. #1 AND #2

Two authors (CM and JR) will independently review all potential studies for inclusion against the eligibility criteria. They will examine the title and abstract and, where necessary, the full text of studies to assess if they are eligible for inclusion. If they cannot reach agreement by discussion, a third author (ME) will make the final decision regarding eligibility.

### Data extraction

Two authors (CM and JR) will independently use a standard form to extract study characteristics and outcome data from the studies. Discrepancies will be checked against the original data. A third author (ME) will make the final decision if there is a disagreement. CM will enter data in RevMan meta-analysis software (RevMan v5.1, 2011). Where possible we will report group outcomes at baseline; preoperatively (following intervention), according to the time at which they were reported during hospital admission (grouped as: under five days postoperatively, five to 10 days postoperatively, over 10 days postoperatively); and at discharge from hospital. Any outcomes measured after discharge from hospital will be grouped as less than one month, one to six months, and over six months.

### Quality assessment

Methodological quality will be assessed using the PEDro scale [28] by a trained assessor (ME). The strength of the recommendations will be summarized using the GRADE approach [29]. Scores will be based on all information available from both the published version and the authors themselves. No eligible trial will be excluded on the basis of poor quality.

### Data analysis

For binary (dichotomous) outcome measures, we aim to calculate a pooled estimate of treatment effect for each outcome across studies using risk ratio where appropriate and a corresponding 95% confidence interval (CI). For continuous outcome measures, we will calculate a pooled estimate of treatment effect by calculating the mean difference and the corresponding 95% CI, or the standardized mean difference and 95% CI wherever the data to be pooled for a single outcome are reported using different measurement tools. When analyzing count data, a decision will be made whether to treat these as dichotomous, continuous, time-to-an-event or as a rate depending on whichever of these methods allows the greatest number of data points to be included in the meta-analysis. We plan to analyze time-to-event data using the hazard ratio and 95% CI. In the event of missing, incomplete or unclear data we plan to contact the original investigators. If we do not obtain the necessary data for analysis, we will describe the study results in the text.

We plan to assess the degree of heterogeneity between studies using the I^2^ statistic. This measure describes the percentage of total variation across studies that is caused by heterogeneity rather than by chance. The values of I^2^ lie between 0% and 100%, and a simplified categorization of heterogeneity that we plan to use is low (I^2^ value of less than 25%), moderate (I^2^ value of between 25% and 50%), and high (I^2^ value of over 50%) [30]. If sufficient studies are included, we will assess reporting bias among the studies using the funnel plot method discussed in the Cochrane Handbook for Systematic Reviews of Interventions [30]. If asymmetry is present, we will explore possible causes including publication bias, methodological quality and true heterogeneity. We will enter data extracted from included studies into RevMan software (RevMan v5.1, 2011). If there is no significant heterogeneity, we will compute pooled estimates of the treatment effect for each outcome under a fixed-effect model. If there is significant heterogeneity, we will compute pooled estimates of the treatment effect for each outcome using a random-effects model. If there is significant heterogeneity (over 50%) and there are sufficient studies included in the review, we will investigate the possible causes further by performing the following subgroup analyses: strength regimens (for example, ≥30% MIP); endurance regimens (for example <30% MIP); presence of respiratory muscle weakness at baseline; duration of training prior to surgery; type of surgery, that is, cardiac, thoracic, upper abdominal (excluding bariatric), bariatric.

Some of these subgroups have been considered in previous systematic reviews that examined IMT [31] or that had PPCs as an outcome [32].

### Sensitivity analysis

We will test the robustness of our results through sensitivity analyses excluding unpublished studies, small studies (n <10), studies with a PEDro score less than 5, studies using inspiratory resistive flow training and studies that apply resistive training without controlling flow.

## Discussion

This review aims to provide the best available evidence of the effects of preoperative IMT on postoperative outcomes in patients who are undergoing open cardiothoracic or upper abdominal surgery. This will inform physiotherapists, respiratory therapists, surgeons and others working in the perioperative setting of the value of these interventions.

## Abbreviations

CI: confidence interval; PPC: postoperative pulmonary complication; IMT: inspiratory muscle training; MIP: maximal inspiratory pressure.

## Competing interests

The authors declare that they have no competing interests.

## Authors’ contributions

JR and CM wrote the first draft of the protocol. CG reviewed the outcome measures for relevance to patients. ME drafted the search strategies. All authors contributed to revision of the protocol. All authors read and approved the final manuscript.
